# Interleukin‐5 alleviates cardiac remodelling via the STAT3 pathway in angiotensin II‐infused mice

**DOI:** 10.1111/jcmm.18493

**Published:** 2024-07-04

**Authors:** Caijie Shen, Nan Wu, Xiaomin Chen, Jianye Peng, Mingjun Feng, Jian Wang, Yibo Yu

**Affiliations:** ^1^ Department of Cardiovascular Medicine The First Affiliated Hospital of Ningbo University Ningbo China; ^2^ Cardiovascular Medicine The Second Affiliated Hospital of University of South China Hengyang China

**Keywords:** angiotensin II, cardiac remodelling, interleukin‐5, macrophage differentiation, STAT3 pathway

## Abstract

Interleukin‐5 (IL‐5) has been reported to be involved in cardiovascular diseases, such as atherosclerosis and cardiac injury. This study aimed to investigate the effects of IL‐5 on cardiac remodelling. Mice were infused with angiotensin II (Ang II), and the expression and source of cardiac IL‐5 were analysed. The results showed that cardiac IL‐5 expression was time‐ and dose‐dependently decreased after Ang II infusion, and was mainly derived from cardiac macrophages. Additionally, IL‐5‐knockout (IL‐5−/−) mice were used to observe the effects of IL‐5 knockout on Ang II‐induced cardiac remodelling. We found knockout of IL‐5 significantly increased the expression of cardiac hypertrophy markers, elevated myocardial cell cross‐sectional areas and worsened cardiac dysfunction in Ang II‐infused mice. IL‐5 deletion also promoted M2 macrophage differentiation and exacerbated cardiac fibrosis. Furthermore, the effects of IL‐5 deletion on cardiac remodelling was detected after the STAT3 pathway was inhibited by S31‐201. The effects of IL‐5 on cardiac remodelling and M2 macrophage differentiation were reversed by S31‐201. Finally, the effects of IL‐5 on macrophage differentiation and macrophage‐related cardiac hypertrophy and fibrosis were analysed in vitro. IL‐5 knockout significantly increased the Ang II‐induced mRNA expression of cardiac hypertrophy markers in myocardial cells that were co‐cultured with macrophages, and this effect was reversed by S31‐201. Similar trends in the mRNA levels of fibrosis markers were observed when cardiac fibroblasts and macrophages were co‐cultured. In conclusions, IL‐5 deficiency promote the differentiation of M2 macrophages by activating the STAT3 pathway, thereby exacerbating cardiac remodelling in Ang II‐infused mice. IL‐5 may be a potential target for the clinical prevention of cardiac remodelling.

## 1 INTRODUCTION

Chronic heart failure (CHF) is a complex clinical syndrome that can be caused by many pathological factors, such as hypertension, pressure load and heart diseases.^1^ CHF is one of the most important causes of death worldwide, especially in economically underdeveloped areas.^2^ A large number of studies have shown that the process of CHF is often accompanied by the occurrence of cardiac remodelling, and delaying the progression of cardiac remodelling can significantly improve cardiac dysfunction and improve patient prognosis.^1^, ^2^, ^3^

Interleukins (ILs) are pluripotent cytokines secreted by both immune cells and non‐immune cells, and several ILs have been reported to be involved in cardiac remodelling. In a recent study, down‐regulation of IL‐6 expression was reported to significantly alleviate cardiac hypertrophy and fibrosis in mice with transverse aortic constriction.^4^ In another study, IL‐10 knockout was found to significantly increase pressure overload‐induced cardiac remodelling by inhibiting bone marrow fibroblast progenitor cell homing and differentiation to fibroblasts.^5^ Deletion of IL‐12p35, the common subunit of IL‐12 and IL‐35, increased the differentiation of CD4+ T lymphocyte‐dependent type 2 macrophages, thereby exacerbating cardiac fibrosis through the P65 pathway in angiotensin II (Ang II)‐treated mice.^6^ In animal studies, IL‐18 has been reported to successfully exacerbate cardiac remodelling induced by osteopontin, Ang II or pressure overload.^7^, ^8^, ^9^ IL‐22‐neutralizing antibodies significantly reduced Ang II‐induced cardiac inflammation and alleviated cardiac hypertrophy and cardiac dysfunction.^10^

IL‐5, a member of the IL‐2 superfamily, is secreted by immune cells such as macrophages and lymphocytes.^11^ IL‐5 can regulate the STAT pathway, mediate downstream signalling and participate in a variety of disease processes.^12^ IL‐5 has also been shown to be involved in a variety of cardiovascular diseases. Previous studies have reported that increased IL‐5 expression can significantly inhibit both Ang II‐ and high‐fat diet‐induced atherosclerosis in mice, suggesting that IL‐5 may be a regulatory target for atherosclerosis.^13^, ^14^, ^15^ However, the role of IL‐5 in cardiac remodelling remains unknown. In this study, Ang II was used to build cardiac remodelling model and IL‐5‐knockout (IL‐5−/−) mice were used to determine the effects of IL‐5 on cardiac remodelling. In order to determine whether IL‐5 is involved in the regulation of cardiac remodelling through the STAT3 pathway, S31‐201 was used to inhibit the STAT3 pathway phosphorylation and observe the progress of cardiac remodelling.

## 2 MATERIALS AND METHODS

### 2.1 Animals and animal models

Both IL‐5−/− mice and wild‐type (WT) mice with a C57BL/6J background were purchased from the Institute of Model Zoology of Nanjing University. All mice were housed in the animal room of Wuhan University People's Hospital, and male mice aged 9 weeks were used in this study because low degree of cardiac remodelling was observed in female mice.^16^ First, WT mice were infused with Ang II (1000 ng/kg/min, ENZO) for different times or infused with different Ang II concentrations for 14 days, and control mice were infused with saline. Then, cardiac IL‐5 expression in each group was measured. Additionally, both WT mice and IL‐5−/− mice were infused with Ang II (1000 ng/kg/min) or saline for 14 days, and body weight (BW) and heart weight (HW) measurements were obtained. Furthermore, both WT mice and IL‐5−/− mice were infused with Ang II and treated daily with DMSO, or S31‐201 (2.5 mg/kg, Sigma).^17^ Among which, S31‐201 is the specific inhibitor of STAT3 pathway and inhibits STAT3 activation by inhibiting complex formation, STAT3 tyrosine phosphorylation and DNA binding. Each group contained 10 mice.

### 2.2 Chronic Ang II infusion

Osmotic minipumps (Alzet, Model 2002) were implanted to infuse Ang II as described in a previous study.^18^ Briefly, the mice were anaesthetised by inhalation of 2% isoflurane, and then the skin on the nape of the neck was cut open after disinfection. The osmotic minipumps containing Ang II (1000 ng/kg/min) were implanted, and the skin was sutured.

### 2.3 Detection of cardiac function

After infusion with Ang II for 14 days, the mice were anaesthetised and then laid flat on the operating table. The hair on the left chest was shaved, and the coupler was applied evenly. Finally, echocardiography was performed using a MyLab™ 30CV ultrasound system (Esaote SpA) equipped with a 10 MHz linear array ultrasound transducer to examine the cardiac function and structure of each mouse, and the left ventricle (LV) end‐diastolic diameter (LVEDD), LV end‐systolic diameter (LVESD), LV ejection fraction (EF), and fractional shortening (FS) measurements were collected and analysed.

### 2.4 Western blot analysis

Total protein was collected after the LV tissue or macrophages (Møs) were lysed. Then, the protein samples were quantitated, separated by electrophoresis and transferred to Immobilon‐FL PVDF membranes (Millipore, USA). After being blocked with 3% foetal bovine serum, the cells were incubated with anti‐α‐SMA, anti‐collagen I, anti‐collagen III, anti‐TGF‐β, anti‐t‐STAT3, anti‐p‐STAT3, anti‐arginase‐1 (Arg‐1) and anti‐GAPDH (all from Abcam or GeneTex) antibodies at 4°C overnight. After being incubated with the secondary antibodies at room temperature for 1 hour, the blots were scanned and analysed.

### 2.5 Quantitative polymerase chain reaction (RT‐qPCR)

Left ventricle tissue or cells were extracted with TRIZOL reagent, and then the total RNA of each sample was collected and reverse transcribed to synthesize cDNA using a reverse transcription kit according to the manufacturer's instructions. Then, PCR amplification was performed using LightCycler 480 SYBR green master mix (Roche) to measure the mRNA expression of target genes, including IL‐5, atrial natriuretic peptide (ANP), B‐type natriuretic peptide (BNP), β‐myosin heavy chain (β‐MHC), α‐SMA, collagen I, collagen III, Arg‐1, CD163, CD206, TGF‐β, IL‐4, IL‐10 and IL‐13. All mRNA expression was normalized to that of GAPDH, and the RT‐qPCR primer sequences are shown in Table 1.

**TABLE 1:** Primers used in this study.

Gene	Forward	Reverse
IL‐5	CACCGAGCTCTGTTGACAAG	TCCTCGCCACACTTCTCTTT
ANP	CCTGTGTACAGTGCGGTGTC	AAGCTGTTGCAGCCTAGTCC
BNP	CTCAAGCTGCTTTGGGCACAAGAT	AGCCAGGAGGTCTTCCTACAACAA
β‐MHC	TCTACCCAGCCAAGATCAAAGT	CCCATTCCTAATAAGCTGTGTGG
α‐SMA	TCCTGACGCTGAAGTATCCGATA	GGCCACACGAAGCTCGTTAT
Collagen I	GCTCCTCTTAGGGGCCACT	CCACGTCTCACCATTGGGG
Collagen III	CTGTAACATGGAAACTGGGGAAA	CCATAGCTGAACTGAAAACCACC
Arg‐1	AACACGGCAGTGGCTTTAACC	GGTTTTCATGTGGCGCATTC
CD36	ATGGGCTGTGATCGGAACTG	TTTGCCACGTCATCTGGGTTT
CD163	TCCACACGTCCAGAACAGTC	CCTTGGAAACAGAGACAGGC
CD206	CAGGTGTGGGCTCAGGTAGT	TGTGGTGAGCTGAAAGGTGA
TGF‐β	TGTTAAAACTGGCATCTGA	GTCTCTTAGGAAGTAGGT
IL‐4	ACGAGGTCACAGGAGAAGGGA	AGCCCTACAGACGAGCTCACTC
IL‐10	ATAACTGCACCCACTTCCCA	GGGCATCACTTCTACCAGGT
IL‐13	CGCAAGGCCCCCACTAC	TGGCGAAACAGTTGCTTTGT
GAPDH	AACTTTGGCATTGTGGAAGG	CACATTGGGGGTAGGAACAC

### 2.6 Enzyme‐linked immunosorbent assay (ELISA)

Serum was collected after the blood samples were centrifuged at 3000 × g for 15 min, and then the serum IL‐5 level in each group was measured using mouse IL‐5 ELISA kits (Thermo Fisher) according to the manufacturer's instructions. All samples were measured in duplicate.

### 2.7 Histological analysis

Mouse hearts were isolated, fixed with formaldehyde, embedded in paraffin, cut to a thickness of approximately 5 μm and mounted onto slides. Then, wheat germ agglutinin (WGA) staining was performed for histopathological analysis, and more than 200 cells in each group were used to analyse the cross‐sectional areas (CSAs) of myocardial cells (MCs). Picrosirius red (PSR) staining was used to determine the LV collagen expression levels. Immunofluorescence staining was used to measure cardiac IL‐5 expression. Double immunofluorescence staining using anti‐F4/80 and anti‐IL‐5 antibodies, anti‐F4/80 and anti‐p‐STAT3 antibodies or anti‐F4/80 and anti‐CD206 antibodies was used to identify the presence of IL‐5 in Møs, the expression of p‐STAT3 in Møs and M2 Møs. Fluorescence intensity was analysed as the expression of target protein.

### 2.8 Cell study and treatment

Bone marrow‐derived Møs were isolated from both WT mice and IL‐5−/− mice as described in a previous study.^19^, ^20^ Briefly, male mice aged 7–8 weeks were euthanized, and the tibias were isolated in a sterile environment. Then, both segments were opened, and the cells were flushed out from the lumen using RPMI‐1640 medium. After the red blood cells were lysed, the cells were rinsed with PBS and centrifuged to obtain monocytes (Monos). The isolated cells were treated with M‐CSF (50 ng/mL) for 8 days to promote differentiation into Møs.^19^, ^20^ Mouse MCs and cardiac fibroblasts (CFs) were purchased from ATCC, and all cells were cultured in RPMI‐1640 medium and treated as follows:

First, Møs (10^6^ cells) and MCs (10^6^ cells) were co‐cultured and divided into the following 8 groups: 1: MCs + WT Møs; 2. MCs + IL‐5−/− Mø; 3. MCs + WT Mø + S31‐201 (10 μM)^21^; 4. MCs + IL‐5−/− Mø + S31‐201; 5: MCs + WT Mø + Ang II (100 nM)^18^; 6. MCs + IL‐5−/− Mø + Ang II; 7. MCs + WT Mø + S31‐201 + Ang II; and 8. MCs + IL‐5−/− Mø + S31‐201 + Ang II. After treatment for 24 hours, the MCs were collected for ANP and BNP mRNA analysis.

Additionally, Møs and CFs (10^6^ cells) were co‐cultured and divided into 8 groups as described above. The mRNA expression of α‐SMA, collagen I, and collagen III in CFs was measured.

### 2.9 Statistical analysis

All data in the present study are expressed as the mean ± standard deviation (SD) and were analysed using GraphPad Prism 7. Student's *t*‐tests were used to compare the differences between two groups, and one‐way or two‐way ANOVA, followed by Tukey's multiple comparisons test, was performed to compare the differences between more than two groups. A value of *p* < 0.05 was considered to be statistically significant.

## 3 RESULTS

### 3.1 Ang II infusion reduced IL‐5 expression in cardiac macrophages in mice

**FIGURE 1:**
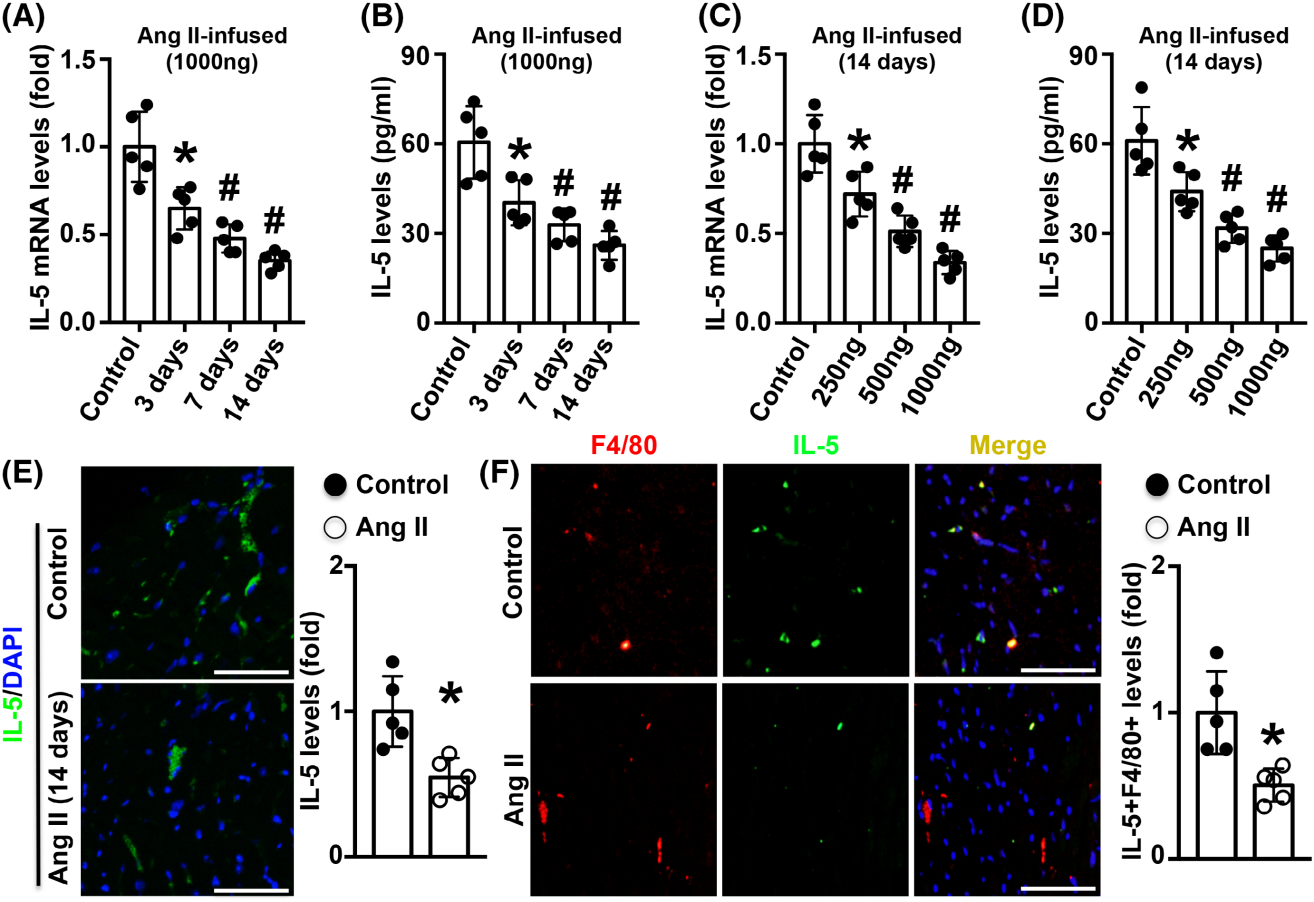
Effects of Ang II infusion on IL‐5 expression. (A, B) Cardiac IL‐5 mRNA expression and serum IL‐5 levels were determined after Ang II infusion for different times. (C, D) The expression of cardiac IL‐5 and serum IL‐5 was measures after treatment with different doses of Ang II. (E) Cardiac IL‐5 levels (green signal) in the control and Ang II groups were analysed by immunofluorescence staining. (F) Double immunofluorescence staining of anti‐F4/80 (red signal) and anti‐IL‐5 (green signal) in Ang II‐infused mice, and the target signal was yellow. Scale bar = 50 μm, *N* = 5 in each group. *p < 0.05 versus the control group. ^#^ *p* < 0.05 versus the previous time or previous dose group.

Ang II infusion time‐dependently reduced both cardiac IL‐5 mRNA expression and serum IL‐5 levels (Figure 1A,B). Similar trends were observed when mice were infused with different Ang II concentrations for 14 days (Figure 1C,D). Immunofluorescence staining showed that Ang II infusion for 14 days significantly reduced cardiac IL‐5 expression compared with that of the control group (Figure 1E). Double staining with anti‐F4/80 and anti‐IL‐5 antibodies also showed that cardiac Mø‐derived IL‐5 levels were reduced by Ang II infusion (Figure 1F).

### 3.2 IL‐5 deficiency worsened cardiac remodelling and dysfunction in Ang II‐treated mice

**FIGURE 2:**
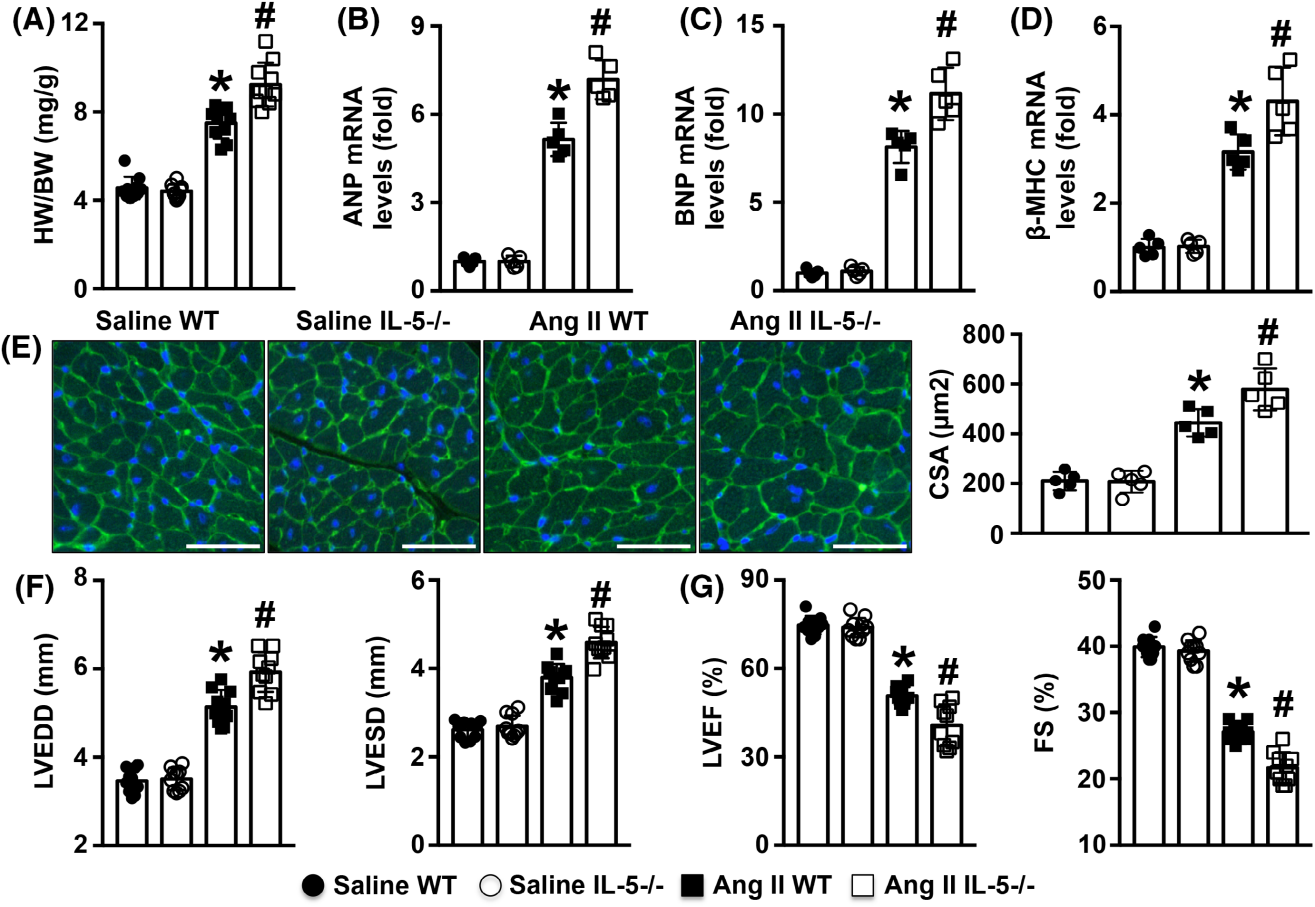
Effects of IL‐5 knockout on Ang II‐induced cardiac hypertrophy and cardiac dysfunction. (A). The HW/BW ratios were analysed; *N* = 10 in each group. (B–D). mRNA expression of ANP, BNP and β‐MHC in the LV in the four groups; *N* = 5 in each group. (E). WGA staining and the myocyte CSA in each group; more than 200 cells were analysed. (F, G). The LVEDD, LVESD, LVEF and EF were determined by echocardiography; *N* = 10 in each group. Scale bar = 50 μm, *p < 0.05 versus the saline WT group. ^#^ *p* < 0.05 versus the Ang II WT group. ANA, atrial natriuretic peptide; BNP, B‐type natriuretic peptide; BW, body weight; CSA, cross‐sectional areas; EF, ejection fraction; FS, fractional shortening; HW, heart weight LVEDD, end‐diastolic diameter; LVESD, LV end‐systolic diameter. WGA, wheat germ agglutinin; WT, Wild‐type.

In Ang II‐infused mice, IL‐5 deficiency increased the ratios of HW/BW (Figure 2A). In addition, the Ang II‐induced increases in the mRNA expression of hypertrophy markers, such as ANP, BNP, and β‐MHC, were mitigated by IL‐5 deletion (Figure 2B–D). The WGA staining results showed that the CSA was increased in Ang II‐infused WT mice and further elevated in Ang II‐infused IL‐5−/− mice (Figure 2E). In addition, IL‐5 deficiency increased both the LVEDD and LVESD while decreasing both the LVEF and FS in Ang II‐treated mice (Figure 2F,G).

### 3.3 IL‐5 knockout exacerbated Ang II‐induced cardiac fibrosis

**FIGURE 3:**
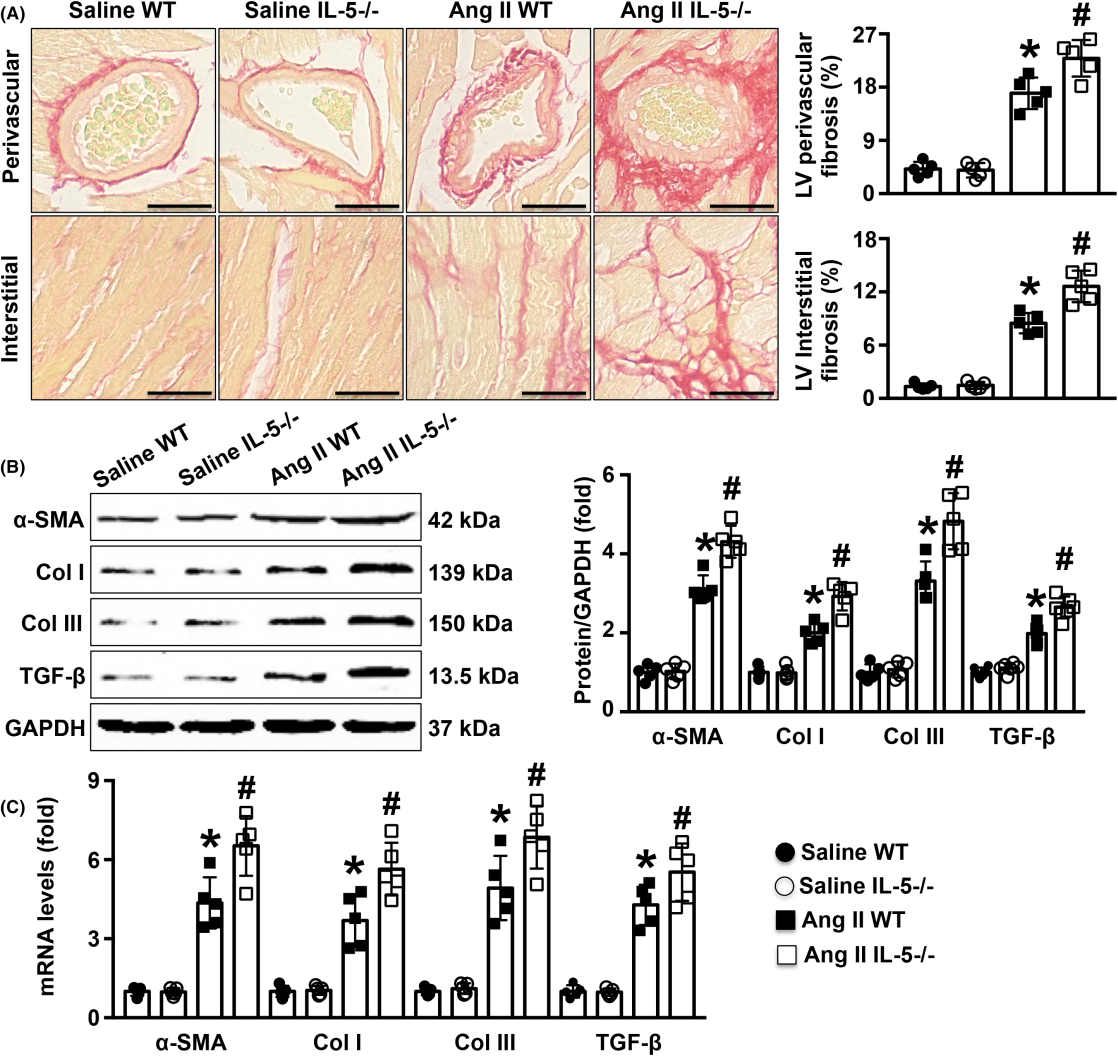
Effects of IL‐5 deletion on Ang II‐induced cardiac fibrosis. (A) The degree of fibrotic area in LV in each group was measured by PSR staining and analysed (400×). (B, C) Protein levels and mRNA expression of α‐SMA, collagen I, collagen III and TGF‐β in the LV in the four groups were measured by western blotting and RT‐qPCR, respectively. Scale bar = 100 μm, *N* = 5 in each group. *p < 0.05 versus the saline WT group. ^#^ *p* < 0.05 versus the Ang II WT group. LV, left ventricle, PSR, picrosirius red; WT, wild‐type.

The PSR staining results showed that the degree of perivascular fibrosis and interstitial fibrosis in LV was significantly higher in the Ang II‐infused IL‐5−/− mice than in the Ang II‐infused WT mice (Figure 3A). Western blot analysis revealed that IL‐5 deletion significantly increased the protein levels of cardiac α‐SMA, collagen I, collagen III and TGF‐β in Ang II‐infused mice (Figure 3B). Similar trends in the mRNA levels of these genes were observed (Figure 3C).

### 3.4 IL‐5 deletion promoted M2 macrophage differentiation in Ang II‐infused mice

**FIGURE 4:**
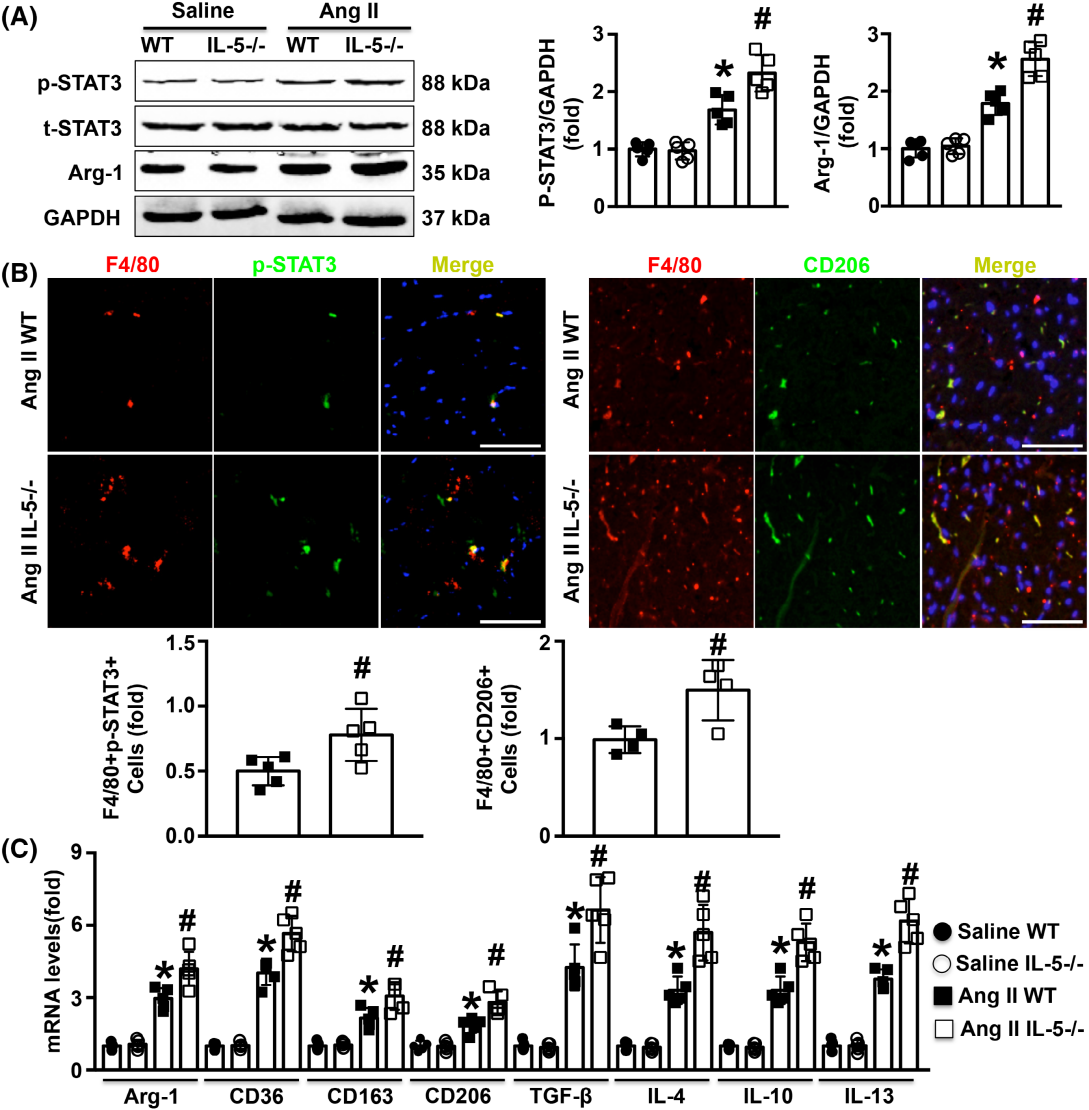
Effects of IL‐5 deficiency on M2 macrophage differentiation in Ang II‐treated mice. (A) The expression of p‐STAT3, t‐STAT3 and Arg‐1 in the LV in the four groups was measured by western blotting. (B) p‐STAT3 protein (green signal) and CD206 protein (green signal) expression in cardiac Møs (red signal), of Ang II WT mice and Ang II IL‐5−/− mice was measured by double immunofluorescence staining, and the target signal was yellow. (C) The mRNA levels of Arg‐1, CD163, CD206, TGF‐β, IL‐4, IL‐10 and IL‐13 in the LV were measured in the four groups. Scale bar = 50 μm, *N* = 5 in each group. *p < 0.05 versus the saline WT group. ^#^ *p* < 0.05 versus the Ang II WT group. LV, left ventricle; WT, wild‐type.

Cardiac STAT3 phosphorylation was measured, and the results showed that both levels of p‐STAT3 and Arg‐1 were significantly increased in Ang II‐infused WT mice and further elevated in Ang II‐infused IL‐5−/− mice (Figure 4A). Increased p‐STAT3 expression was observed in cardiac Møs (Figure 4B). Furthermore, infusion with Ang II increased the number of cardiac M2 Møs, and IL‐5 knockout further promoted M2 Mø differentiation in Ang II‐treated mice (Figure 4B). Similar trends in the mRNA expression of M2 Mø‐related cytokines, including Arg‐1, CD163, CD206, TGF‐β, IL‐4, IL‐10 and IL‐13, were observed (Figure 4C).

### 3.5 S31‐201 treatment alleviated cardiac remodelling and cardiac dysfunction in Ang II‐infused IL‐5−/− mice

**FIGURE 5:**
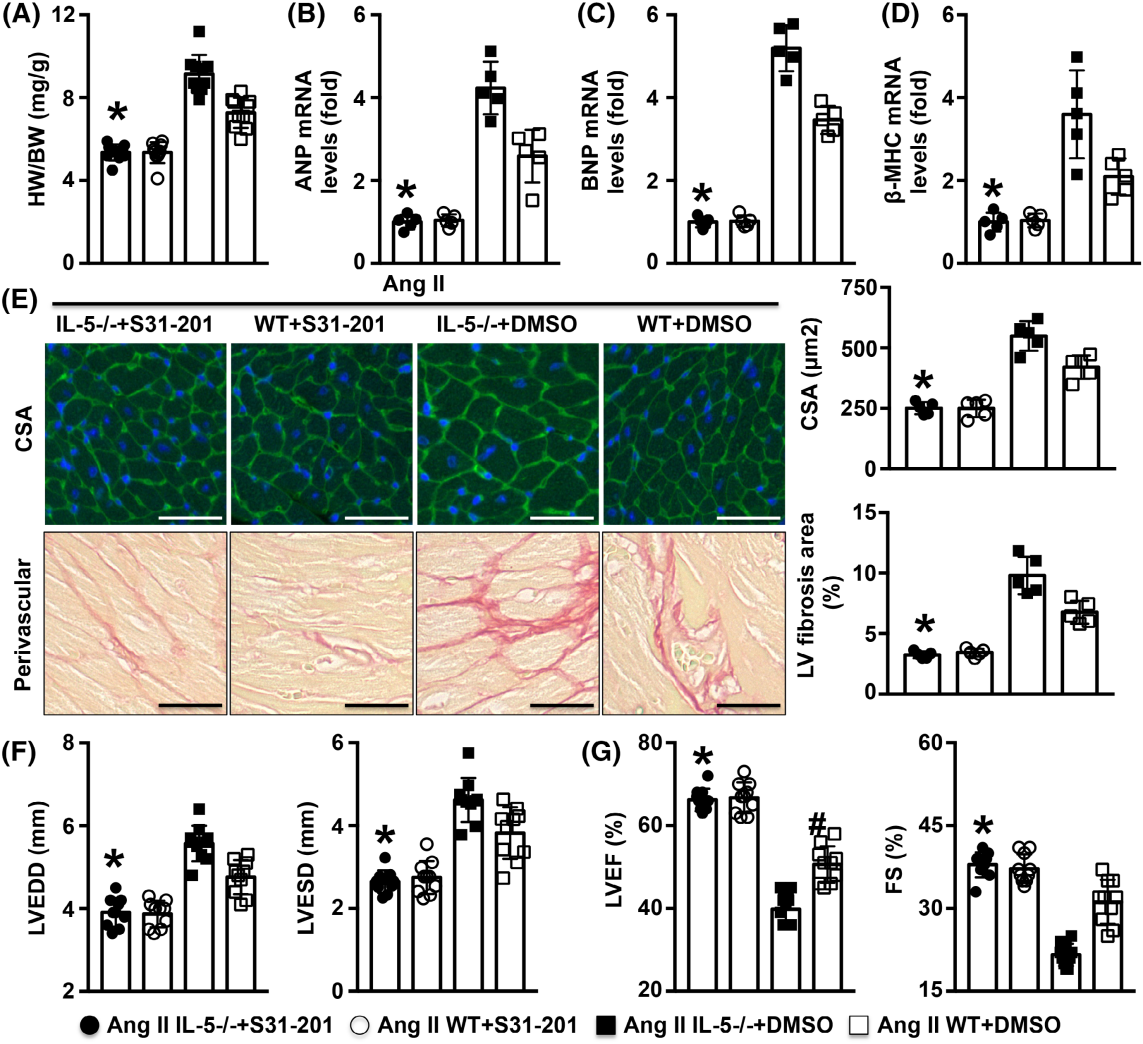
Effects of S31‐201 on cardiac remodelling in Ang II IL‐5−/− mice. (A) The HW/BW ratios were measured in the four groups. (B–D). The mRNA expression of ANP, BNP and β‐MHC for each group were measured. (E) Myocyte CSA and cardiac fibrosis in the LV were measured. (F, G) The LVEDD, LVESD, LVEF and FS in each group were determined by echocardiography. Scale bar = 50 μm, *N* = 5–10 in each group. *p < 0.05 versus the Ang II IL‐5−/− + DMSO group. ANA, atrial natriuretic peptide; BNP, B‐type natriuretic peptide; BW, body weight; CSA, cross‐sectional areas; FS, fractional shortening; HW, heart weight LVEDD, end‐diastolic diameter; LVESD, LV end‐systolic diameter.

Treatment with S31‐201 significantly reduced the HW/BW ratios, and downregulated mRNA levels of ANP, BNP and β‐MHC in Ang II‐infused IL‐5−/− mice (Figure 5A–D). In addition, both the CSA and the degree of collagen disposition were decreased by S31‐201 treatment in Ang II‐infused IL‐5−/− mice (Figure 5E). Furthermore, lower the LVEDD and LVESD and higher LVEF and FS were observed in Ang II‐infused mice when S31‐201 was given (Figure 5F,G).

### 3.6 Treatment with S31‐201 reversed M2 macrophage differentiation in Ang II‐infused IL‐5−/− mice

**FIGURE 6:**
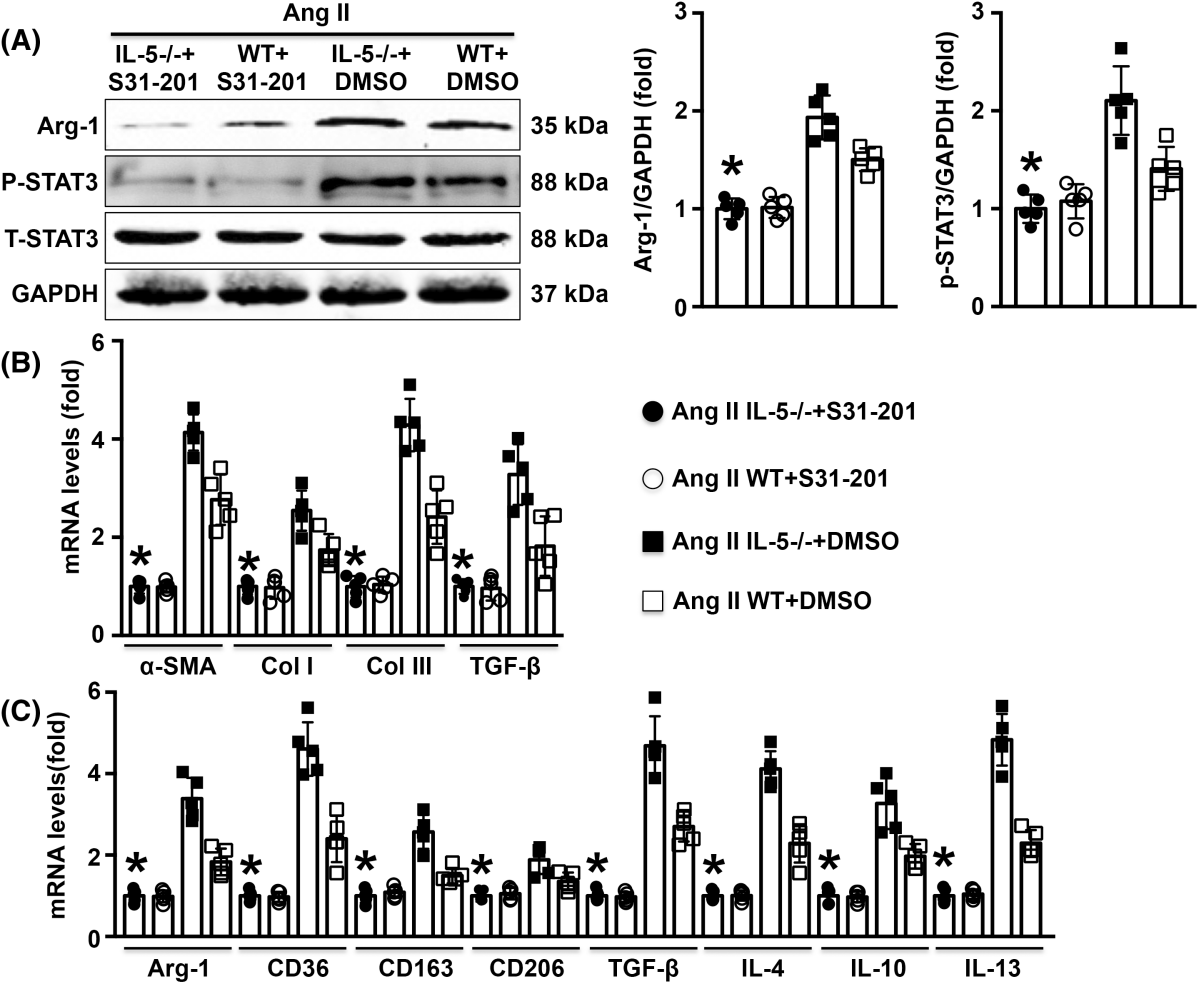
Effects of S31‐201 on M2 macrophage differentiation in Ang II IL‐5−/− mice. (A) Cardiac Arg‐1, p‐STAT3 and t‐STAT3 levels were measured and analysed in each group. (B, C) The mRNA expression levels of α‐SMA, collagen I, collagen III, TGF‐β, Arg‐1, CD163, CD206, TGF‐β, IL‐4, IL‐10 and IL‐13 in the LV were measured in the four groups. *N* = 5 in each group. *p < 0.05 versus the Ang II IL‐5−/− + DMSO group. LV, left ventricle.

Western blot analysis showed that treatment with S31‐201 significantly reduced both Arg‐1 expression and STAT3 phosphorylation in Ang II‐infused IL‐5−/− mice (Figure 6A). In addition, mRNA expression levels of cardiac α‐SMA, collagen I, collagen III and TGF‐β were decreased by S31‐201 in Ang II‐infused mice (Figure 6B). Similar trends in the mRNA levels of Arg‐1, CD163, CD206, TGF‐β, IL‐4, IL‐10 and IL‐13 were observed (Figure 6C).

### 3.7 IL‐5 deletion exacerbated Ang II‐induced myocardial cell hypertrophy and cardiac fibroblast collagen deposition in vitro

**FIGURE 7:**
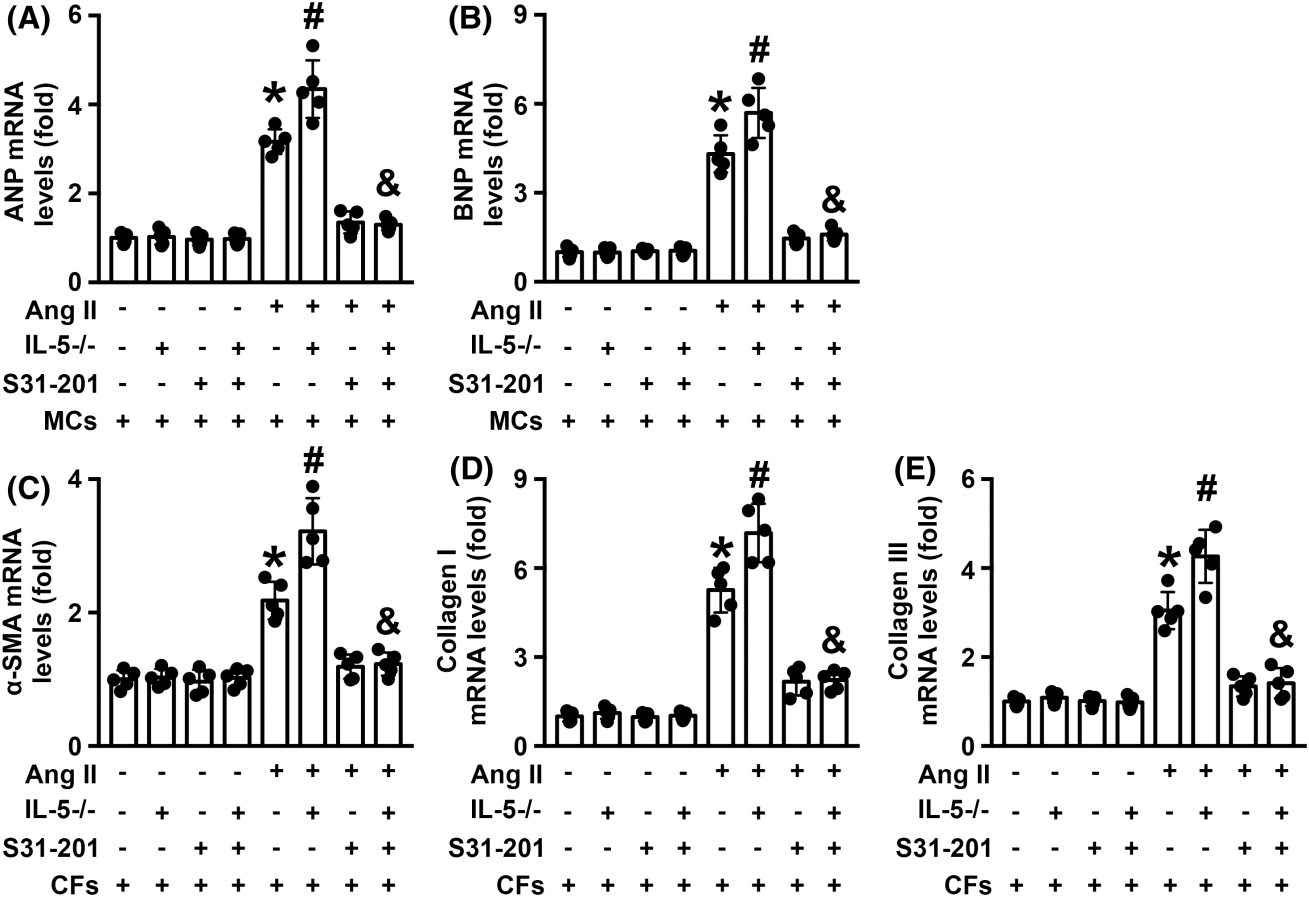
Effects of macrophage IL‐5 knockout on Ang II‐induced myocardial cell hypertrophy and collagen synthesis in cardiac fibroblasts. (A, B) ANP and BNP mRNA levels in MCs and (C–E) α‐SMA, collagen I and collagen III mRNA levels in CFs were measured. *N* = 5 in each group. *p < 0.05 versus the MCs/CFs + WT Mø group. ^#^ *p* < 0.05 versus the MCs/CFs + WT Mø + Ang II group. ^&^ *p* < 0.05 versus the MCs/CFs + IL‐5−/− Mø + Ang II group. ANA, atrial natriuretic peptide; BNP, B‐type natriuretic peptide; CFs, cardiac fibroblasts; MCs, myocardial cells; WT, wild‐type.

In MCs co‐cultured with WT Møs, both ANP and BNP mRNA levels were increased by Ang II treatment. The ANP and BNP mRNA levels of MCs were further elevated when the cells were co‐cultured with IL‐5−/− Møs, but these effects were significantly reversed by S31‐201 treatment (Figure 7A,B). Similar trends in the mRNA levels of α‐SMA, collagen I and collagen III were observed when CFs were co‐cultured with WT Møs (Figure 7C–E).

## 4 DISCUSSION

In the present study, our data demonstrated for the first time that Ang II treatment significantly decreased IL‐5 expression, which was secreted mainly by macrophages. IL‐5 deficiency promoted the development of cardiac hypertrophy, exacerbated the degree of cardiac fibrosis and further worsened cardiac dysfunction in Ang II‐infused mice. More p‐STAT3 protein expression in macrophages and M2 macrophage differentiation were observed in Ang II‐infused IL‐5−/− mice than in Ang II‐infused WT mice. When STAT3 phosphorylation was blocked by S31‐201, these effects mediated in IL‐5−/− mice were significantly reversed. In addition, similar results were also observed in vitro.

Data from both clinical experiments and animal studies have shown that IL‐5 expression is significantly altered in cardiovascular disease. Decreased IL‐5 expression has been observed in human CHF, high‐fat diet‐induced mouse atherosclerosis and doxorubicin‐induced mouse acute cardiac injury.^13^, ^14^, ^15^, ^22^ Ang II is the joint link of renin‐angiotensin system and the most important signal mediator. After bind to the receptors of target cell, especially AT1, Ang II lead to the activation of a variety of immune cells and the release of inflammatory substances, fibroblast proliferation, the production of intracellular free radicals, tissue and organ damage, mitochondrial dysfunction and other pathological processes. Therefore, it is widely used in the study of hypertension and cardiac remodelling. In this study, we found that Ang II, an important inflammatory substance that induces cardiac remodelling, time‐ and dose‐dependently decreased cardiac IL‐5 expression, which suggests that reduced IL‐5 expression may be associated with Ang II‐induced cardiac remodelling. Although both immune and non‐immune cells are sources of IL‐5, IL‐5 is rarely secreted by non‐immune cells and is mainly secreted by immune cells such as lymphocytes, macrophages and dendritic cells.^13^, ^15^, ^23^, ^24^ Our results showed that Ang II treatment significantly reduced the IL‐5 expression derived from cardiac macrophages. These results suggest that the reduction in macrophage‐derived cardiac IL‐5 may play an important role in Ang II‐induced cardiac remodelling.

To investigate the role of IL‐5 in cardiac remodelling, IL‐5−/− mice were used to establish a mouse model of cardiac remodelling using Ang II infusion. The results showed that IL‐5 deficiency significantly increased the mRNA expression of cardiac hypertrophy markers, such as ANP, BNP and β‐MHC. Cardiac fibrosis is a serious pathological change associated with the process of cardiac remodelling, and it is also an important factor that reflects cardiac remodelling. We also determined the effect of IL‐5 deletion on cardiac fibrosis, and the results showed that IL‐5 knockout also exacerbated collagen deposition and elevated the mRNA expression of various markers of cardiac fibrosis. These results confirmed that IL‐5 knockout significantly exacerbated Ang II‐induced cardiac remodelling. Previous studies have found that IL‐5 does not affect blood pressure in Ang II‐infused mice, suggesting that the regulation of IL‐5 in cardiac remodelling is independent of blood pressure.

Numerous studies have shown that multiple immune cell types are involved in the process of cardiac remodelling, such as macrophages, monocytes and dendritic cells.^25^, ^26^ Among them, the relationship between macrophages and pressure overload‐induced cardiac remodelling has been the most widely studied.^24^, ^25^, ^26^ Macrophages can be divided into M1 macrophages and M2 macrophages, among which M1 macrophages lead to tissue injury by releasing inflammatory factors such as IL‐6 and TNF‐α, while M2 macrophages mediate repair of injury tissue by releasing inflammatory factors such as IL‐4 and TGF‐β. In cardiac remodelling, M1 macrophages begin to rise in the early stages, while M2 macrophages decrease in the early stages of immune response and subsequently increase in feedback, and elevated M2 macrophages promote M1 macrophage mediated repair of myocardial cells. The essence of M2 macrophage in repairing the heart is that the damaged myocardial cells are replaced by fibrous tissue.^9^, ^18^, ^27^, ^28^ However, this repair effect is not perfect. While stabilizing the heart structure, it inevitably damages the systolic and diastolic functions of the heart, causing or exacerbating cardiac remodelling and CHF.^6^, ^9^, ^18^, ^27^

Numerous studies have demonstrated that the STAT3 pathway is one of the important signalling pathways regulating macrophage differentiation into the M2 phenotype.^18^, ^29^ IL‐5 has been widely showed to regulate macrophage differentiation in a variety of micro‐environments.^15^, ^30^, ^31^, ^32^ Considering that STATs are the most important signalling pathways associated with IL‐5, we hypothesized that IL‐5 regulates macrophage differentiation into the M2 phenotype through the STAT3 pathway. To confirm this hypothesis, we first examined the phosphorylation of STAT3 pathway factors and found a significant increase in p‐STAT3 expression in both the heart and cardiac macrophages in Ang II‐infused IL‐5−/− mice compared with Ang II‐infused WT mice. Next, we examined the differentiation of M2 macrophages and the expression of M2‐related markers and inflammatory cytokines. The results showed that more M2 macrophages, as well as M2 macrophage‐related markers and cytokines, were observed in Ang II‐infused IL‐5−/− mice. These results suggest that IL‐5 deletion promotes the M2 differentiation of macrophages by activating the STAT3 pathway, thus exacerbating the progression of cardiac remodelling in Ang II‐infused mice.

To further confirm the above hypothesis, a specific STAT3 pathway inhibitor, which has been demonstrated to significantly decrease STAT3 phosphorylation, was used in this study. The results showed that treatment with S31‐201 significantly decreased the expression of cardiac hypertrophic markers such as ANP, BNP and β‐MHC, as well as cardiac fibrosis markers such as α‐SMA, collagen I, collagen III, TGF‐β. In addition, the values of LVEDD and LVESD were decreased, while the values of LVEF and FS were increased by S31‐201 in Ang II‐infused IL‐5−/− mice. These results suggested that S31‐201 reversed cardiac remodelling in Ang II‐infused IL‐5−/− mice. In addition, decreased mRNA expression levels of M2 macrophage‐related markers and cytokines were found in S31‐201‐treated Ang II‐infused IL‐5−/− mice compared with DMSO‐treated Ang II‐infused IL‐5−/− mice. These results further support our hypothesis and demonstrate that the regulation of IL‐5 deletion on Ang II‐induced cardiac remodelling is mediated by the STAT3 signalling pathway.

To exclude the influence of interactions between body factors and further verify these conclusions, myocardial cells and cardiac fibroblasts were individually co‐cultured with macrophages. The results showed that co‐culture with IL‐5−/− macrophages significantly promoted Ang II‐induced myocardial cell hypertrophy, which was markedly reversed by S31‐201 treatment, and similar trends in fibrosis‐related cytokine mRNA expression in cardiac fibroblasts were observed. These results also suggest that IL‐5 regulates Ang II‐mediated cardiac hypertrophy and cardiac fibrosis through the STAT3 pathway in vitro, which is consistent with the conclusions of our animal experiments.

Previous studies have found that IL‐5 can regulate cardiac remodelling, such as improving cardiac remodelling after myocardial infarction and exacerbating cardiac remodelling induced by hypereosinophilia.^33^, ^34^ However, the regulatory role of IL‐5 in Ang II‐induced cardiac remodelling has not been reported. Base on this study, we found for the first that IL‐5 expression is reduced in cardiac remodelling, and the decreased expression of IL‐5 further activates the STAT3 signalling pathway, which is closely related to cardiac M2 macrophages polarization, the occurrence of cardiac remodelling and the deterioration of cardiac function. The down‐regulation of IL‐5 expression caused by various risk factors is closely related to the process of cardiac remodelling.

## 5 CONCLUSIONS

Our study confirmed that knockout of IL‐5 promote the phosphorylation of STAT3 in macrophages, thereby promoting M2 macrophage differentiation and exacerbating Ang II‐induced cardiac remodelling and cardiac dysfunction. IL‐5 is an important target for the clinical prevention of cardiac remodelling and delaying the progression of CHF.

## AUTHOR CONTRIBUTIONS

**Xiaomin Chen:** Writing – review and editing (supporting). **Caijie Shen:** Conceptualization (equal); investigation (equal); methodology (equal); software (equal). **Nan Wu:** Investigation (equal); methodology (equal); writing – original draft (equal). **Jianye Peng:** Investigation (equal); software (equal). **Mingjun Feng:** Validation (equal); writing – original draft (equal). **Jian Wang:** Supervision (equal); visualization (equal). **Yibo Yu:** Writing – original draft (equal).

## CONFLICT OF INTEREST STATEMENT

The authors declare no potential conflict of interest.

## Data Availability

The datasets used and analysed during the current study are available from the corresponding author on reasonable request. All data generated or analysed during this study are included in this published article.
